# MORC2 regulates C/EBPα-mediated cell differentiation via sumoylation

**DOI:** 10.1038/s41418-018-0259-4

**Published:** 2019-01-15

**Authors:** Jia Liu, Qing Zhang, Banlai Ruan, Wei Chen, Jianyu Zheng, Buxuan Xu, Peijia Jiang, Zhifeng Miao, Feng Li, Jessie Yanxiang Guo, Liu Cao, Guiling Wang

**Affiliations:** 1 Department of Cell Biology, Key Laboratory of Cell Biology, Ministry of Public Health and Key Laboratory of Medical Cell Biology, Ministry of Education, China Medical University, Shenyang, 110122 China; 2 Department of Surgical Oncology and General Surgery, First Hospital of China Medical University, Shenyang, 110001 China; 3 Division of Medical Oncology, Rutgers Cancer Institute of New Jersey, New Brunswick, NJ 08903 USA

**Keywords:** Oncogenes, Prognostic markers

## Abstract

The expression and activity of CCAAT/enhancer-binding protein α (C/EBPα) are involved in sumoylation modification, which is critical to divert normal cells from differentiation to proliferation. However, the role and underlying mechanism of C/EBPα in cancer is poorly understood. Human MORC2 (microrchidia family CW-type zinc-finger 2), is a member of the MORC proteins family containing a CW-type zinc-finger domain. Here, we found that MORC2 interacted with TE-III domain of C/EBPα, and the overexpression of MORC2 promoted wild-type C/EBPα sumoylation and its subsequent degradation, which didn’t significantly observe in mutant C/EBPα-K161R. Furthermore, the overexpression of MORC2 inhibited C/EBPα-mediated C2C12 cell differentiation to maintain cell cycle progression. Moreover, the striking correlation between the decreased C/EBPα expression and the increased MORC2 expression was also observed in the poor differentiation status of gastric cancer tissues. Most notably, the high expression of MORC2 is correlated  with an aggressive phenotype of clinical gastric cancer and shorter overall survival of patients. Taken together, our findings demonstrated that MORC2 expression regulated C/EBPα-mediated the axis of differentiation/proliferation via sumoylation modification, and affected its protein stability, causing cell proliferation and tumorigenesis.

## Introduction

The tumorigenesis is a multistep process with numerous changes in cell proliferation, differentiation, and/or survival [1]. The molecular alterations that occurs during this progression include the over-expression and/or amplification of transcription factors, growth factors, and their receptors, or the silencing of tumor suppressive gene. CCAAT/enhancer-binding proteins (C/EBPs) are one such group of transcription factors that control cellular proliferation and differentiation in a variety of tissues [2, 3], which are involved in a variety of different cell process including physiology and pathology.

The C/EBPα is a founding member of the C/EBP proteins family, containing a highly conserved transactivation domain (TE-I, TE-II, and TE-III) and a basic leucine zipper (bZip) domain [4]. Given evidence suggests that transcription factors are modification by SUMO [5], which affects protein–protein interactions [6], sub-cellular localization [7], transcriptional activity [8], and protein stability [5]. Modification of C/EBPα by SUMO-1 at lysine reside 161 lies within the TE-III domain, which enables binding to specific proteins to affect its level of the sumoylation [8–10]. In addition, sumoylation and ubiquitin are conjugated either sequentially or simultaneously to functionally regulate the substrate protein in many cases [5, 11]. Sumoylation is oftentimes deregulated in cancer, and the sumoylation of C/EBPα decreases the tumor suppressive activity [10, 12], which is associated with various human cancers [3, 13, 14], suggesting that loss of C/EBPα might be associated with the switch from a cell differentiation to a cell proliferation program in cancer [15], but the mechanism of the down-regulation of C/EBPα in cancer remains elusive.

Human MORC2 (microrchidia family CW-type zinc-finger 2), containing a CW-type zinc-finger and three coiled-coil domains, is a member of the MORC proteins family and mainly localizes in the nucleus [16, 17]. Recent studies revealed that MORC2 repressed gene transcription [17, 18] promoted chromatin remodeling during the DNA-damage response [18] and regulated lipogenesis [19]. Our research indicated that MORC2 promoted gastric cancer cell proliferation and tumorigenesis [20, 21].

Here, we found that MORC2 interacted with TE-III domain of C/EBPα,which be related to the sumoylation of C/EBPα. Moreover, the increased MORC2 expression negatively correlates with the decreased C/EBPα, which was also shown in the differentiation status of gastric cancer samples. These results open a new line of study on the underlying mechanism of down-regulated C/EBPα expression in cancer, and identify that MORC2 plays an important role in control C/EBPα-mediated the axis of differentiation/proliferation, which is involved in tumorigenesis.

## Results

### MORC2 interacts with TE-III domain of C/EBPα

**Fig. 1:**
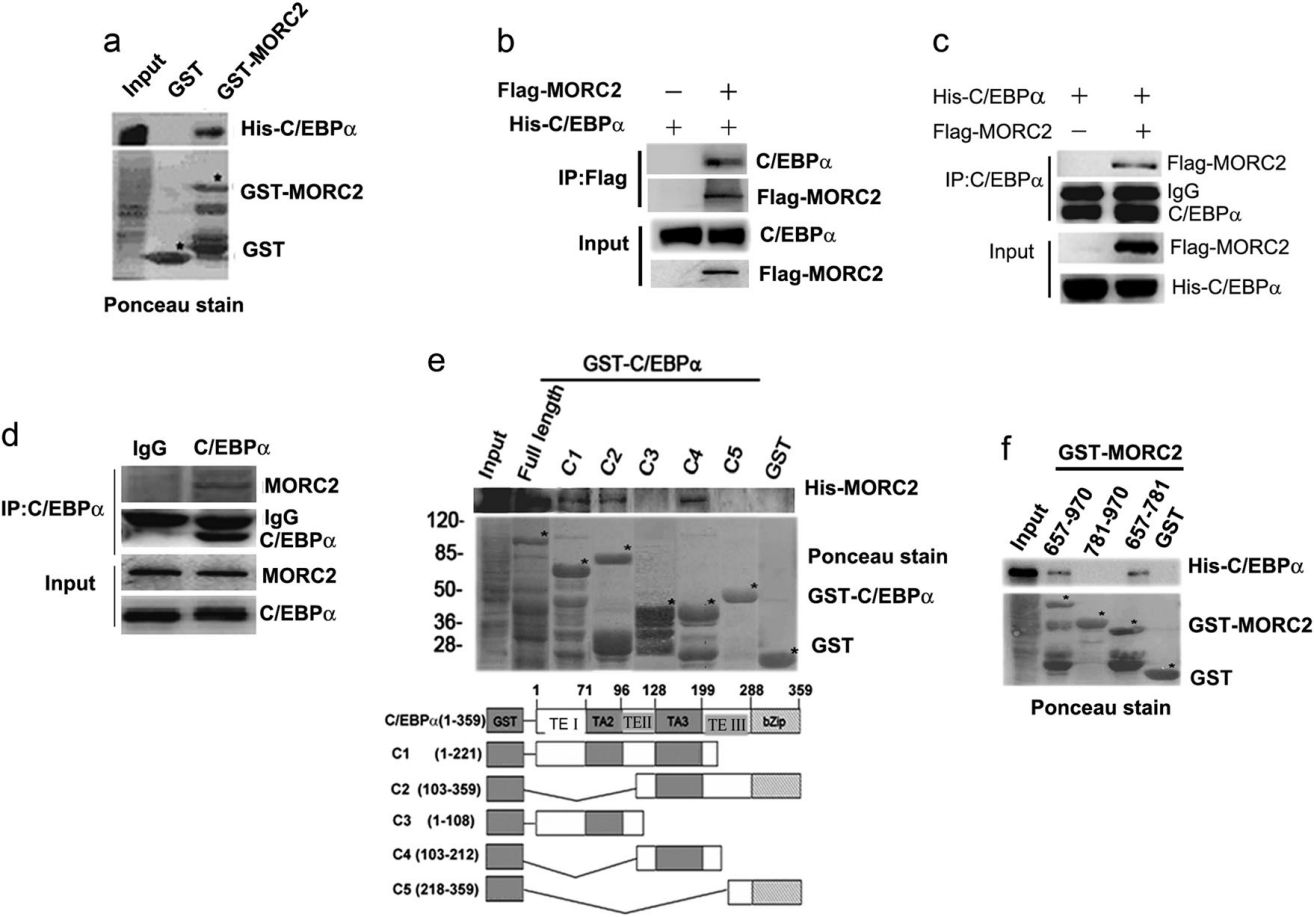
MORC2 interacts with TE-III domain of C/EBPα. **a** MORC2 directly binds C/EBPα in vitro. In vitro–transcribed and translated His-C/EBPα were incubated with either GST-MORC2 or GST. Bound proteins were analyzed by western blot with His-tag antibody. Black stars indicated the GST or GST-MORC2. **b**, **c** MORC2 interacts with C/EBPα in co-IP assay. HEK-293T cells were transiently transfected with His-C/EBPα plus Flag-MORC2 plasmids for 36 h, as indicated. Total lysates were subjected to immunoprecipitate and Western Blot with Flag-tagged or His-tagged antibody, as indicated. **d** The interaction between endogenous C/EBPα and MORC2 was examined by IP assay in vivo. **e**, **f** Mapping of binding domains in C/EBPα and MORC2. For GST pull-down assay, GST, GST-C/EBPα or GST-MORC2 deletions were incubated with indicated proteins transcripted and translated in vitro. Bound proteins were detected with indicated antibodies. Black stars indicated the GST-fusion proteins

An assay using GST-MORC2 and in vitro translated C/EBPα demonstrated specific interaction between these two proteins (Fig. 1a). To further confirm MORC2 interacting with C/EBPα in vivo, a reciprocal co-immunoprecipitation assay was performed. As can be seen in Fig. 1b–d, C/EBPα was co-precipitated with MORC2 in vivo, suggesting that C/EBPα was specifically interacted with MORC2. A series of purified GST-fused C/EBPα proteins corresponding to the truncation and deletion constructs were performed by GST pull-down assays, and indicated that in vitro-translated MORC2 bound to the full length GST-C/EBPα, GST-C1, GST-C2, GST-C4 but not GST-C3, GST-C5 or GST alone, showing that in vitro-translated MORC2 could bind to the GST-C4 (103–212) of C/EBPα (Fig. 1e), which is within TE-III domain [4]. Vice versa, in vitro-translated C/EBPα protein specifically binds to amino acids 657–781 of GST-MORC2 (Figure 1f). Taken together, these data indicate that MORC2 specifically binds to TE-III domain of C/EBPα.

### MORC2 negatively regulates C/EBPα expression

**Fig. 2:**
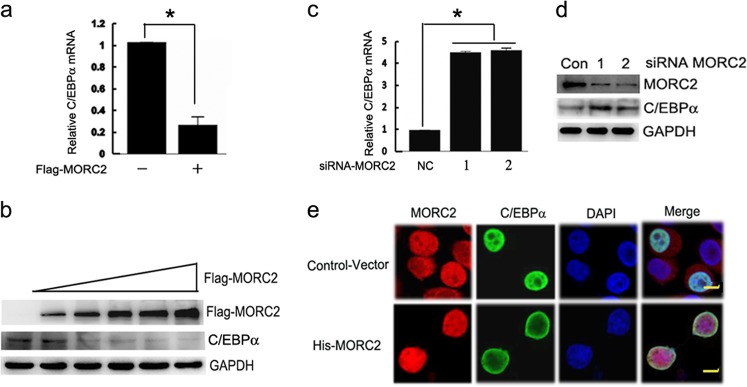
MORC2 negatively regulates C/EBPα expression. **a**, **b** The mRNA and protein levels of C/EBPα  were reduced by the enhancing  ecotopic MORC2 expression. A dose dependent increase of MORC2 plasmids were transfected into SGC-7901 cells. QPCR and Western blot assays were performed to detect the mRNA and protein levels of endogenous C/EBPα. **c**, **d** Knockdown of MORC2 can increase the mRNA and protein levels of C/EBPα. BGC-823 cells were transfected with siRNAs targeting MORC2 or non-silencing control. After 30 h of transfection, the mRNA and protein levels of C/EBPα were measured by qPCR and western blotting assays. (**e**) Co-localization of C/EBPα and MORC2 was observed by confocal microscopy. SGC-7901 cells were transfected with His-C/EBPα along with pcDNA3.1-vector or pcDNA3.1-MORC2 and performed immunofluorescence assays with specific MORC2 and C/EBPα antibodies. Alexa Fluor 488(Green) and Alexa Fluor 546 (red) were used to detect C/EBPα (green) and MORC2 (red) respectively. Yellow indicates co-localization. Blue, DNA dyed DAPI

In order to examine whether MORC2 regulates the expression of C/EBPα, we transfected with His-MORC2 in the SGC-7901 cells and found that the mRNA and protein levels of C/EBPα were reduced with the increased ecotopic MORC2 expression (Fig. 2a, b). Whereas, specific knockdown of MORC2 up-regulated the mRNA and protein level of C/EBPα (Fig. 2c, d) through targeting siRNA MORC2 or non-silencing control in the BGC-823 cells. In addition, we got the similar results with overexpressing of MORC2 in SGC-7901 cells or knock down of MORC2 in BGC-823 cells (supplementary Figure1a and 1b). Together, these results indicate that MORC2 can specifically represses C/EBPα expression.

In addition, immunofluorescence assays found that MORC2 co-localized with C/EBPα in the nucleus when MORC2 expression was normal (Fig. 2e, upper panel). Interestingly, the nuclear localization of C/EBPα was less when MORC2 was overexpressed (Fig. 2e, second lane in the lower panel), the co-localization of C/EBPα and MORC2 was mainly seen in nuclear membrane (Fig. 2e, last lane in the lower panel). Therefore, overexpression of MORC2 may lead to the decreased nuclear localization of C/EBPα, which may be related to the sumoylation of C/EBPα.

### MORC2 overexpression promotes the sumoylation of C/EBPα and its subsequent degradation

**Fig. 3:**
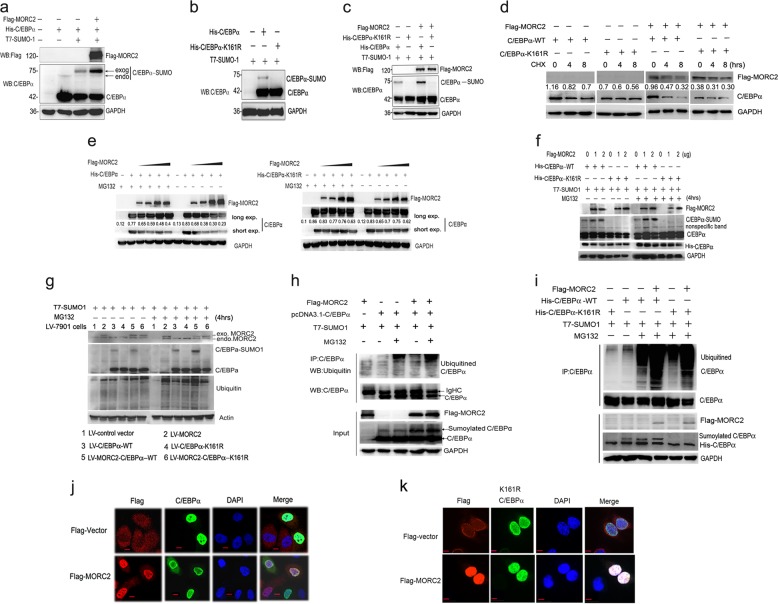
MORC2 overexpression promotes the sumoylation of C/EBPα and its subsequent degradation. **a** The sumoylation level of C/EBPα was increased by the ecotopic MORC2. HEK-293TT cells were co-transfected with His-C/EBPα, Flag-MORC2, and T7-SUMO1. Total lysates were subjected to western blot with indicated antibodies. **b** The deficient sumoylation mutant vector of His-C/EBPα-K161R was identified. HEK-293T cells were co-transfected with T7-SUMO1 and wild type of His-C/EBPα or SUMO deficient mutant of His-C/EBPα-K161R. Total lysates were subjected to western blot with indicated antibodies. **c** The overexpressed MORC2 increased the sumoylation level of C/EBPα, while didn’t affect the mutant C/EBPα-K161R level. HEK-293T cells were transfected with the expression vectors for Flag-MORC2, T7-SUMO1 and His-C/EBPα or His-C/EBPα-K161R, as indicated. Total lysates were subjected to western blot with indicated antibodies. **d** A cycloheximide (CHX) chase is carried out to really prove that MORC2 affects the stability of the wild-type and not K161R CEBP/α. We co-transfected wild-type His-C/EBPα or mutant C/EBPα-K161R and Flag-MORC2 into HEK-293T cells with the cycloheximide (CHX) treatment. After treatment with cycloheximide (CHX) (50 μg/ml) for 4 and 8 h, cell lysates were subjected to western blot with indicated antibodies. **e** The increased MORC2 affects wild-type not K161 mutant of C/EBPα stability. HEK-293T cells were transfected with the expression vectors for Flag-MORC2 and His-C/EBPα or His-C/EBPα-K161R, as indicated. After incubation with MG132 (10 μM) for 4 h, cell lysates were subjected to western blot with indicated antibodies. **f** MORC2 affects the sumoylated C/EBPα and its stability in HEK-293T cells. HEK-293T cells were transfected T7-SUMO1 and these vectors, as indicated in Fig. 3e, cell lysates were subjected to western blot with indicated antibodies. **g** MORC2 promotes the sumoylated C/EBPα levels and its stability in stable expressing SGC-7901 cell lines. These lentivirus-mediated cells were transfected with T7-SUMO1 and HA-ub, as indicated. After incubation with MG132 (10 μM) for 4 h, cell lysates were subjected to western blot with indicated antibodies. **h**, **i** IP assays demonstrate that MORC2 promotes the sumoylation of C/EBPα and its subsequent degradation in the presence of MG132. HEK-293T cells (**h**) and stable expressing SGC-7901 cell lines (**i**) were transfected with T7-SUMO1, as indicated. After incubation with MG132 (10 μM) for 4 h, cell lysates were subjected to IP assays with C/EBPα antibody, western blot detected with indicated antibodies. **j** The nuclear localization of C/EBPα was observed by confocal microscopy when MORC2 was overexpression. SGC-7901 cells were transiently co-transfected with His-C/EBPα and Flag-MORC2 for 36 h, as indicated, and incubated with Flag-tagged antibody to detect MORC2 expression, specific C/EBPα antibody to detect His-C/EBPα expression. Alexa Fluor 488 (green) and Alexa Fluor 546 (red) were used to detect His-C/EBPα and Flag-MORC2 (red) respectively. Yellow indicates co-localization. Blue, DNA dyed DAPI. **h** The nuclear localization of C/EBPα-K161R was observed by confocal microscopy when MORC2 was overexpression. SGC-7901 cells were transiently co-transfected with His-C/EBPα-K161R and Flag-MORC2 for 36 h, and incubated with Flag-tagged antibody to detect MORC2 expression, specific C/EBPα antibody to detect His-C/EBPα-K161R expression. Alexa Fluor 488 (green) and Alexa Fluor 546 (red) were used to detect His-C/EBPα-K161R and Flag-MORC2 (red) respectively. Yellow indicates co-localization. Blue, DNA dyed DAPI

The above results indicated that MORC2 interacted with TE-III domain of C/EBPα, which overlaps with the SUMO-1 conjugation lysine residue 161 site [4]. Thus, we tried to elucidate whether the reduction of C/EBPα by MORC2 was related to its sumoylation. To test this postulation, we co-transfected His-C/EBPα, T7-SUMO-1 and Flag-MORC2 into HEK-293T cells (which lacks endogenous C/EBPα) and showed that the level of sumoylated C/EBPα was enhanced (Fig. 3a). The deficient sumoylation mutant vector of His-C/EBPα-K161R was constructed to further study the effect of MORC2 on the sumoylation of C/EBPα (Fig. 3b). As can be seen in Fig. 3c, the sumoylation of C/EBPα at K161 was enhanced when MORC2 was co-expressed (Fig. 3c, second lane, column 3 compared to column 1), while no sumoylation of C/EBPα was detected when MORC2 was co-transfected with the SUMO-deficient mutant of C/EBPα-K161R (Fig. 3c, column 4 of second lane).

To clarify the reason why MORC2 down-regulates C/EBPα expression and whether sumoylation affects its subsequent degradation by the proteasome. As shown in the second lane of Fig. 3d, compared to without CHX treatment, the protein levels of wild-type C/EBPα were largely decreased, while the protein levels of mutant C/EBPα-K161R haven’t been significantly affected by CHX treatment. To strengthen this point, we performed these assays with the stable co-expressing SGC-7901 cell lines (LV-MORC2-C/EBPα-WT and LV-MORC2-C/EBPα-K161R) and got the same results, indicating that the protein levels of C/EBPα-K161R were more stable than C/EBPα-WT with increasing CHX treatment time (supplementary Figure 1c). These results proved that MORC2 affects the protein stability of the wild-type but not mutant K161R C/EBPα. Meanwhile, the protein level of C/EBPα was decreased with increasing amounts of MORC2 in HEK-293 cells without MG132 (a specific inhibitor of the proteasome) incubation, while treatment with MG132 largely increased the protein level of C/EBPα (Fig. 3e, left panel). However, the protein levels of mutant C/EBPα-K161R had no significant change with the enhancing ectopic MORC2 expression even with MG132 treatment (Fig. 3e, right panel)). In addition, overexpressing MORC2 increased the sumoylation levels of wild-type C/EBPα but not C/EBPα-K161R mutant in the presence of T7-sumo1 (Fig. 3f, second lane, column 2 and 3 compared to column 1). MG132 treatment increased sumoylation levels of wild-type C/EBPα (Fig. 3f, second lane, column 8 and 9 compared to column 2 and 3) compared to C/EBPα-K161R in the presence of MG132 and MORC2. The same results with lentiviral vectors of SGC-7901 cells were indicated that MORC2 promoted the sumoylation of C/EBPα-WT (Fig. 3g, second lane, column 5 compared to column 3), and higher sumoylation levels of C/EBPα-WT showed it undergoes rapid degradation compared with MG132 treatment (Fig. 3g, second lane, column 11 compared to column 5). IP assays further indicated that the sumoylated C/EBPα had an increase of ubiquitined C/EBPα expression in the presence of MORC2 and MG132 (Fig. 3h, right column 1 compared to column 2 of first lane and Fig. 3i, left column 4 compared to column 3 of first lane). C/EBPα-K161R had less ubiquitin level than C/EBPα-WT in the presence of MG132 and MORC2 (Fig. 3I, first lane,left column 6 compared to column 4), suggesting that MORC2 promotes the sumoylation of wild-type C/EBPα and its subsequent degradation.

In addition, the immunofluorescence assays showed that the nuclear localization of C/EBPα was less (Fig. 3j, column 2 of down panel) compared to C/EBPα-K161R (Fig. 3k, column 2 of down panel) when MORC2 was overexpressed. The confocal results are consistent with the above (Fig. 2e, down panel). Therefore, these results further indicate that overexpression of MORC2 induces the decreased nuclear localization of C/EBPα and be related to the sumoylation of C/EBPα.

### MORC2 overexpression inhibits C/EBPα-mediated C2C12 cell differentiation

**Fig. 4:**
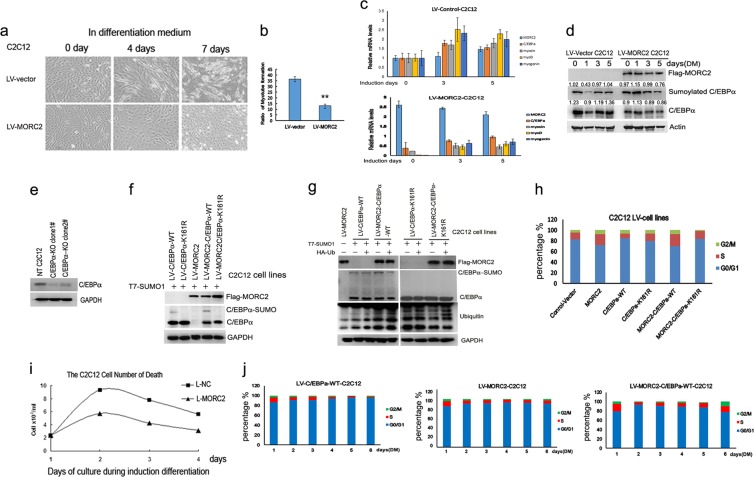
MORC2 regulates C/EBPα-mediated C2C12 muscle cell differentiation. **a** In differentiation medium MORC2 overexpressing C2C12 cells keep proliferating and do not start differentiation. Phase-contrast microphoto of C2C12 and LV-MORC2 differentiation cells at 0, 4, and 7 days of differentiation. Original magnification 10×. To induce skeletal muscle differentiation, when reaching 70–80% confluence, cells of infection with Lentivirus Flag-control vector (LV-NC) and Lentivirus Flag-MORC2 (LV-MORC2) were cultured in DM. DM was changed every 2 days and differentiation was completed in 7 days. **b** The ratios of myotube formation in LV-MORC2 cells (13.0% ± 1.5%) on day 6 of differentiation showed significant less than LV-control vector cells (36.4% ± 2.2%) *P* < 0.0001. **c** With the process from C2C12 myoblast to mature myocytes triggered by 2% horse serum, muscle-related protein molecules expression changed prior to simulation. The differentiating C2C12 myoblast cells was investigated by subjecting confluent C2C12 cells from GM (growth medium) to DM (differentiation medium) to induce differentiation for 7 days, and isolating mRNA extracts at the indicated time point. Real-time PCR detected the mRNA levels of myoD, myogenin, myosin and C/EBPα. The mRNA levels of MyoD (myogenic differentiation antigen), myogenin and myosin increased gradually in LV-control C2C12 cells (“3 group” and “5 group” compared to “0” group in upper panel) compared to “0 day” of differentiation (the mRNA levels of LV-control C2C12 in “0 day” of the differentiation were set to 1 and used as a control), but their expression in LV-MORC2 C2C12 cells showed no significant difference (“3 group” and “5 group” compared to “0” group in lower panel) compared to “0 day” (the mRNA levels of LV-MORC2 C2C12 were the relative ratio to the control vector in “0 day” of the differentiation, “0” group in lower panel). C/EBPα mRNA was decreased in the MORC2 overexpressing C2C12 compared to control C2C12 cells in “0 day” (Fig. 4c, lower panel compared to upper panel of “0 group”), but the C/EBPα mRNA expressed approximately equivalent levels in “3 days” and “5 days” compared to “0 day” (Fig. 4c, lower panel). **d** The expression levels of C/EBPα and MORC were detected  with LV-MORC2 and control vector C2C12 cells during induction differentiation by western blotting. The differentiating C2C12 myoblast cells was investigated by subjecting confluent C2C12 cells to induce differentiation for 6 days, and isolating protein extracts at the indicated time point. “0 day” shown cells were cultured with growth medium. Total lysate from C2C12 cells undergoing differentiation was prepared on the days indicated and analyzed by immunoblotting with anti-MORC2, anti-C/EBPα and anti-GAPDH. The C/EBPα protein and its sumoylation levels are gradually increased in LV-control vector C2C12 cells (left panel, “5 days” and “3 days” compared to “1 day”), its protein and sumoylation levels were gradually decreased in LV-MORC2-C2C12 cells (right panel, “5 days” and “3 days” compared to “1 day”). **e** The C/EBPα-KO C2C12 cells were identified the knockout endogenous C/EBPα expression by western blot. C2C12 cells were transfected with the human C/EBPα double nickase plasmid (from Santa Cruz Company) and Selected with puromycin (2 g/mL) to product stable expressing C/EBPα-KO C2C12 cells. **f** MORC2 overexpression promotes the sumoylation of wild-type C/EBPα not mutant K161R in stable co-expressing C2C12 cells. These stable expressing of C2C12 cell lines including LV-control vector, LV-MORC2, LV-C/EBPα-WT, LV-C/EBPα-K161R, LV-MORC2-C/EBPα-WT and LV-MORC2-C/EBPα-K161R were transfected into T7-sumo1, western blot detected the sumoylation levels of C/EBPα with C/EBPα antibody. **g** MORC2 promotes the sumoylation of C/EBPα and its subsequent degradation. These stable expressing of C2C12 cell lines as showed in Fig. 4g were transfected into T7-sumo1 and HA-ub, western blot detected the sumoylation level of C/EBPα and its degradation. **h** MORC2 promotes C/EBPα-mediated C2C12 cell cycle transition from G1 to S. Flow cytometry analyzed the cell cycle transition with these stable expressing of C2C12 cell lines including LV-control vector, LV-MORC2, LV-C/EBPα-WT LV-C/EBPα-K161R, LV-MORC2-C/EBPα-WT and LV-MORC2-C/EBPα-K161R. **i** The died cell number of LV-NC and LV-MORC2 cells, cell viability was determined by Trypan blue dye exclusion assay. After differentiation induction, we counted dead cell number—collecting cell medium after 1 up to 4 days of differentiation—and expressed it as a percentage of dead cells, floating in medium, on total cells (sum of dead and viable cells, in suspension and adhering to plate). **j** MORC2 inhibits C/EBPα-mediated C2C12 cell differentiation via maintaining cell cycle progression. Flow cytometry analyzed cell cycle of LV-C/EBPα-WT, LV-MORC2 and LV-MORC2-C/EBPα-WT cells with the increasing differentiation induction days

To study whether the expression of MORC2 was involved in C/EBPα-mediated cell differentiation, we employed the mouse C2C12 cell lines [22] as a differentiation model. The sub-confluent C2C12 cells of LV-NC and LV-MORC2 (supplementary Figure 2a and 2b) were induced with differentiation medium (DM) contained 2% horse serum instead of 10% fetal bovine serum (FBS) growth medium (GM) at the same time. As reported earlier, after differentiation induction, the C2C12 cells of LV-NC began elongation and started to form the multi nucleus myotubes [22, 23]. On the contrary, the C2C12 cells of LV-MORC2 did not respond to the differentiation stimulus, and continued to proliferate until over-confluence (Fig. 4b). With the differentiation process from C2C12 myoblast to mature myocytes triggered by DM, muscle-related protein expression changed prior to simulation. The mRNA levels of MyoD (myogenic differentiation antigen), myogenin and myosin showed no significant difference in LV-MORC2 C2C12 cells compared to LV-control C2C12 cells (Fig. 4c, “3 group” and “5 group” compared to “0” group). Meanwhile, the C/EBPα mRNA expressed approximately equivalent levels in LV-MORC2 C2C12 cells (Fig. 4c, lower panel,“3 days” and “5 days” compared to “0 day”). The C/EBPα protein and its sumoylation levels are gradually decreased in LV-MORC2-C2C12 cells compared to LV-control C2C12 cells (Fig. 4d, “5 days” and “3 days” compared to “1 day”). These results suggest that overexpression of MORC2 could inhibit C2C12 cell differentiation via sumoylation modification.

The C/EBPα-KO C2C12 cells were generated to further analyze whether MORC2 inhibits C/EBPα-mediated C2C12 cell differentiation (Fig. 4e). The lentiviral vectors of C/EBPα-WT or C/EBPα-K161R and MORC2 were infected into the C/EBPα-KO C2C12 cells to construct stably expressing cells. In the presence of T7-sumo1, the sumoylation levels of C/EBPα had stronger band in the LV-MORC2-C/EBPα-WT C2C12 cells than those in LV-C/EBPα-WT C2C12 cells (Fig. 4f, left column 4 compared to column 1), not band was found in either LV-C/EBPα-K161R or LV-MORC2-C/EBPα-K161R cells (Fig. 4f, left column 2 and column 5), indicating that MORC2 promotes the sumoylation of C/EBPα in C2C12 cells. Meanwhile, in the presence of both HA-Ub and T7-sumo1, the decreased sumoylation levels of C/EBPα (Fig. 4g, left column 5 compared to column 4 of second lane) and increased ubiquitin expression (Fig. 4g, left column 5 compared to column 4 of third lane) were found in LV-MORC2-C/EBPα-WT cells compared to alone T7-sumo1, indicating that MORC2 can promote sumoylation of C/EBPα and its subsequent degradation in C2C12 cells.

And then, MORC2-C/EBPα-WT cells showed significant increase in the percentage of S compared to C/EBPα-WT and MORC2-C/EBPα-K161R (Fig. 4h, column 5 compared to column 3 and column 6), indicating that MORC2 promotes C/EBPα-mediated C2C12 cell cycle transition from G1 to S. Furthermore, to analyze the relation between MORC2 overexpression and the axis of differentiation/proliferation, the induction differentiation cells were measured by collecting the dead cells in the DM, the cell death rate indicated that there were not appreciably affected in LV-MORC2 cells compared to LV-control cells with the increasing differentiation induction days (Fig. 4i), suggesting that MORC2 might inhibit C2C12 cell differentiation. Cell cycle analysis indicated that, with the differentiation process, LV-C/EBPα-WT significantly increased in the percentage of G1 phase cells, and significantly decreased in the percentage of S phase cells (Fig. 4j, left panel). There was no significant decrease in the percentage of S phase cells in LV-MORC2 cells (Fig. 4j, middle panel) compared to LV-C/EBPα-WT cells (Fig. 4j, left panel). However, the co-expression of LV-MORC2-C/EBPα-WT C2C12 cells showed a significant increase in the percentage of S phase cells (Fig. 4j, right panel) compared to LV-C/EBPα-WT cells (Fig. 4j, right panel compared to left panel). In summary, these results indicate that overexpression of MORC2 inhibits C/EBPα-mediated C2C12 cell differentiation and maintains cell proliferation.

In addition, the expression of C/EBPα was upregulated in LV-shRNA-MORC2 C2C12 cells (supplementary Figure 2c). The phase-contrast microscopy observed that the differentiation status of the LV-shRNA-MORC2 C2C12 cells was no significant change compared to LV-shRNA-control C2C12 cells, which might be due to less expression of MORC2 in C2C12 cells.

### MORC2 overexpression attenuates C/EBPα-medicated inhibition of cell proliferation

**Fig. 5:**
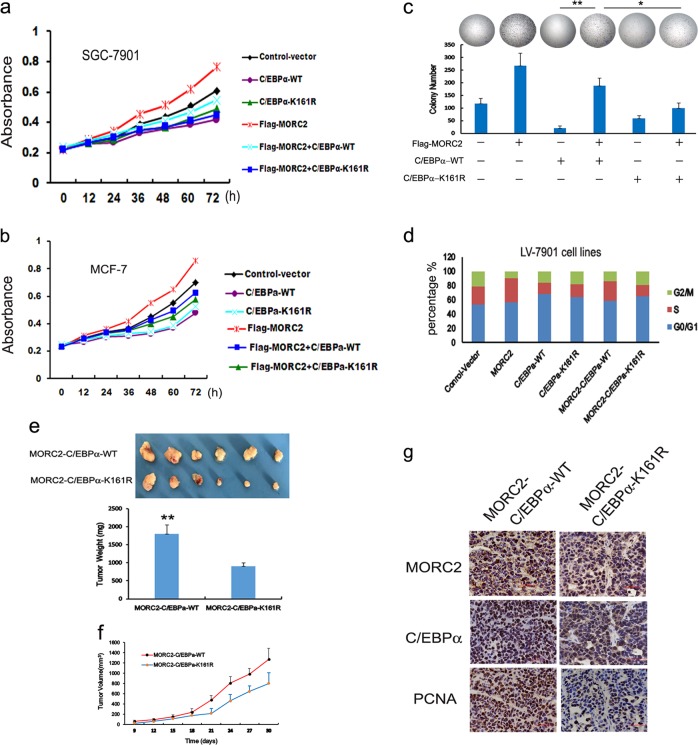
Overexpression of MORC2 attenuates C/EBPα-medicated inhibition of cell proliferation. **a**, **b** The stable expressing Flag-MORC2 of SGC-7901 cells and MCF-7 cells were transiently transfected with His-C/EBPα, and analyzed for cell growth for every 12hrs  by cell counts assay. Values are the means ± SD from three individual experiments. **c** The stable expressing Flag-MORC2 of SGC-7901 cells were transfected with wild type and K161R of His-C/EBPα and performed by colony formation assays. Representative results are shown. **d** Cell cycle analysis indicate that MORC2-C/EBPα-WT not MORC2-C/EBPα-K161R promotes cells transition from G1 to S compared to C/EBPα-WT cells and control vector cells. **e**, **g** MORC2-C/EBPα-WT and MORC2-C/EBPα-K161R cells were injected into right scapular region of the nude mice (*n* = 6 per group). The inoculated mice were terminated in 3 weeks. Each tumor lump was removed from the body. **e** Photographs of tumor weight were quantified. **f** Tumor volumes were measured and data are mean ± SEM. **g** Immunohistochemistry staining of anti-MORC2 and anti-C/EBPa were indicated in nude mice tumor tissues sections, Original magnification, ×400. Degree of intratumoral proliferation was determined by PCNA staining

Emerging evidence shows the critical role of C/EBPα in the regulation of cell growth and cell differentiation [3, 10, 14]. The low level of C/EBPα has been observed in various human cancers [13, 14, 15], suggesting that loss of C/EBPα might be associated to the switch from a cell differentiation to a cell proliferation program in cancer [15]. We, therefore, explored whether MORC2 expression affected on C/EBPα-mediated cell arrest in human cancer. As expected, C/EBPα suppressed cancer cell growth to a greater extent than vector control, while C/EBPα along with Flag-MORC2 attenuated C/EBPα-mediated cancer cell arrest in SGC-7901 cell lines compared to C/EBPα-K161R plus MORC2 (Fig. 5a). We also got a similar result in MCF-7 cell lines (which has almost no endogenous C/EBPα expression) (Fig. 5b), suggesting that the effect of MORC2 on the proliferation and tumorigenesis of cancer cells is independent of C/EBPα expression. In addition, there have significant been evidence to show that the co-expression of MORC2 and C/EBPα-WT formed more colonies (Fig. 5c, left column 4 compared to column 3 and column 6) in vitro and had a significant increase in the percentage of S phase cells (Fig. 5d, left column 5 compared to column 3 and column 6) relative to that seen with C/EBPα-WT and MORC2-C/EBPα-K161R, suggesting that over-expression of MORC2 promotes the growth and cell cycle progression of SGC-7901 via C/EBPα sumoylation.

Subsequently, to further examine the effect of MORC2 and C/EBPα on tumorigenesis in vivo. SGC-7901 cells and MCF-7 cells stably expressing Flag-MORC2 were injected into nude mice via right scapular region. Four weeks after injection, the xenografts tumor was obtained for microscopic histological analysis. The results indicated that the overexpressed MORC2 group developed markedly heavier tumor weight (supplementary Figure 3a), larger volume (supplementary Figure 3b) and accelerated PCNA staining (supplementary Figure 3c, right panel) than vector control group, which is consistent with the result of SGC-7901 cell lines [20], indicating that the increased MORC2 expression and low level of C/EBPα contribute to proliferation and tumorigenesis of cancer cells. After then, we performed the same assays with SGC-7901 cells of co-expression of LV-MORC2-C/EBPα-WT and MORC2-C/EBPα-K161R to explore the effect of MORC2 on C/EBPα-mediated tumorigenesis in vivo. As shown in Fig. 5, the MORC2-C/EBPα-WT group get markedly heavier in tumor weight (Fig. 5e) and larger volume (Fig. 5f), and showed stronger PCNA staining (Fig. 5g, down panel) than MORC2-C/EBPα-K161R group. The formation of xenografts tumor with anti-MORC2 (Fig. 5g, upper panel) and anti-C/EBPα (Fig. 5g, middle panel) staining in nude mice tissues were verified by immunohistochemical analysis. Taken together, these findings suggest that MORC2 overexpression attenuate C/EBPα-medicated inhibition of cell proliferation and tumorigenesis in gastric cancer via sumoylation.

### The increased MORC2 expression negatively correlates with C/EBPα expression and the poor differentiation status of gastric cancer

**Fig. 6:**
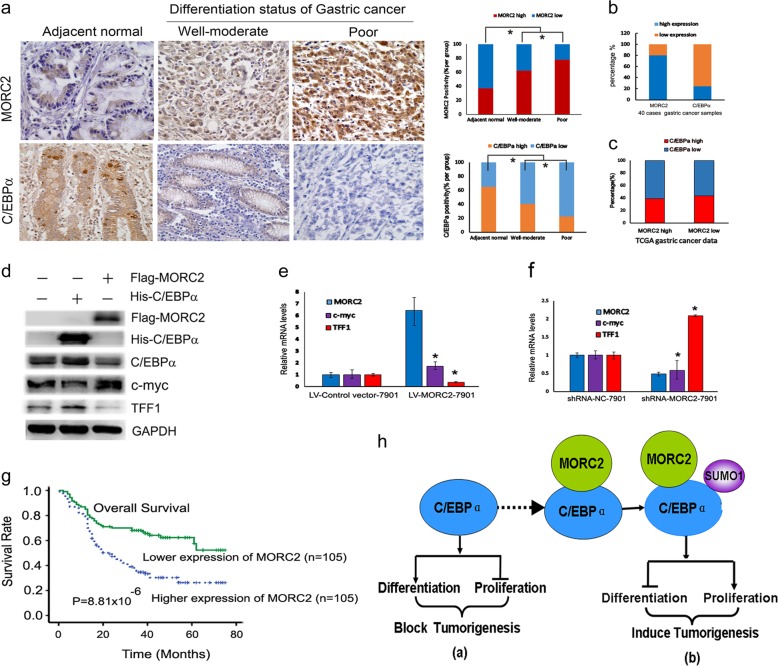
The expression of MORC2 negatively correlates with the differentiation status of gastric cancer. **a** Immunohistochemical  analysis of MORC2 and C/EBPα expression in normal gastric tissues, well-moderate differentiation gastric cancer and differentiation poor gastric cancer tissues. Scale bar, 50 μm. Representative IHC images were shown. The levels of MORC2 and C/EBPα were analyzed in the different differentiation status of gastric cancer samples. **b** The levels of C/EBPα and MORC2 were analyzed with 40 cases of gastric tumor patients. The down-regulation of C/EBPα and up-regulation of MORC2 accounted for ~80% in 40 cases of gastric tumor patients. **c** Low C/EBPα expression accounted for 60% in high expression of MORC2 group. By analyzing the downloaded TCGA gastric cancer public data with expression values of MORC2 and C/EBPα, the expression level in normal tissues and tumor tissues was measured with respective media. Expression value higher than the media is identified as high expression. Vice versa, that is low expression. **d** Western blot detected the protein levels of c-myc and TFF1 when MORC2 or C/EBPα was overexpression in SGC-7901 gastric cancer cells. **e** QPCR  detected the mRNA levels of c-myc and TFF1 when MORC2 was overexpression in SGC-7901 gastric cancer cells. **f** QPCR detected  the  mRNA levels of c-myc and TFF1 when MORC2 was knockdown in SGC-7901 gastric cancer cells. **g** Kaplan–Meier’s analyses illustrated that the 5-year survival rate according to MORC2 protein expression on 210 gastric cancer patients. The lower-expression group was defined as those at or below the median value (1.2-fold), whereas the higher-expression group consisted of patients expressing levels above (1.2-fold). **h** Model of the role of MORC2 in C/EBPα-mediated differentiation and tumorigenesis. In the absent of MORC2, C/EBPα plays a role in cell differentiation promotion and inhibition of cell proliferation, which may block tumorigenesis. While in the present of MORC2 overexpression, MORC2 interacts with C/EBPα and promotes the sumoylation of C/EBPα and its subsequent degradation, which will lead to tumorigenesis

Following, we determined the expression of MORC2 and C/EBPα in gastric cancer tissues and matched adjacent normal tissues from 40 cases gastric tumor patients by performing immunochemical staining of MORC2 and C/EBPα. The increased nuclear staining of MORC2 was observed in poor differentiation gastric cancer compared to well-moderate differentiation and adjacent normal tissues (Fig. 6a, upper panel), poor differentiation gastric cancer tissues with high levels of MORC2 accounted for ~80% of gastric cancer samples (Fig. 6a, upper panel), which showed the striking correlation with decreased C/EBPα (Fig. 6a, down panel). Conversely, the increased C/EBPα expression was shown in adjacent normal tissues and well-moderate differentiation gastric cancer tissues, along with the decreased MORC2 expression (Fig. 6a, down panel). The down-regulation of C/EBPα and up-regulation of MORC2 accounted for ~80% in 40 cases of gastric tumor patients (Fig. 6b). TCGA public data indicated that low C/EBPα expression accounted for 60% in MORC2 high expression group (Fig. 6c). Taken together, the reciprocal expression of low C/EBPα and high MORC2 is associated with maintaining the different differentiation status of gastric cancer.

TFF1, an identified gastric differentiation marker [24], showed to be accompanied by the expression level of C/EBPα[15]. C/EBPα can inhibit c-myc expression, promoting cellular differentiation and growth arrest [25]. Therefore, we further confirmed this point in SGC-7901 gastric cancer cells and obtained the same conclusions, which indicated that the low protein levels of c-myc (Fig. 6d, column 2 of lane 4) and high protein levels of TFF1 (Fig. 6d, column 2 of lane 5) when C/EBPα was overexpression. Meanwhile, we also found that the expression of c-myc was high (Fig. 6d, column 3 of lane 4 and Fig. 6e, column 5) and TFF1 was low (Fig. 6d, column 3 of lane 5 and Fig. 6e, column 6) in the presence of MORC2 overexpression. Conversely, low mRNA level of c-myc (Fig. 6f, column 5) and high level of TFF1 (Fig. 6f, column 6) were shown when MORC2 was knocked out. In summary, these results indicated that MORC2 inhibited C/EBPα-mediated signal, which leads to the switch from a cell differentiation to a cell proliferation program in cancer.

In addition, we studied the correlation between MORC2 expression and clinicopathological characteristics in 210 gastric cancer samples. The results (Table 1, P bold values of *P *< 0.05 indicate statistical significance) showed that high expression of MORC2 was significantly associated with tumor size (*P* = 0.020), poor differentiation (*P* = 0.042), poor depth of invasion (pT) stage (*P* = 0.002), poor distant metastasis (pM) stage (*P* = 0.005) and poor pathological stage (pTNM stage) stage (*P* = 0.021). Moreover, overexpression of MORC2 in clinical gastric cancer was also found to predict shorter overall survival of patients (*P* = 8.81 × 10^−8^; Fig. 6g). These findings suggest MORC2 as a potential prognostic marker and therapeutic target for gastric cancer.

**Table 1:** Comparison of expression levels of MORC2 with clinicopathological features in 210 patients with gastric cancer tissues

	No.	MORC2 fold^a^	*P*-value*
Age (years)			
≥65	82	2.60 (2.00–2.85)	0.389
<65	128	2.45 (2.00–2.75)	
Sex			
Male	158	2.55 (2.00–2.80)	0.103
Female	52	2.35 (1.85–2.75)	
Tumor size (cm)			
≥4	136	2.65 (2.10–2.80)	**0.020**
<4	74	2.35 (1.85–2.75)	
Histological grade			
Low	59	2.35 (1.85–2.75)	**0.042**
High	151	2.55 (2.00–2.80)	
Macroscopic type			
Early stage	30	2.25 (2.00–2.70)	0.382
Borrmann I + II	16	2.70 (2.00–2.825)	
Borrmann III + IV	164	2.55 (2.00–2.80)	
Depth of invasion (pT)			
T1 + T2	171	2.45 (1.90–2.75)	**0.002**
T3 + T4	39	2.70 (2.55–2.85)	
Lymph node status (pN)			
N0 + N1	148	2.375 (1.90–2.80)	0.09
N2 + N3	62	2.65 (2.20–2.80)	
Distant metastasis (pM)			
No	197	2.45 (2.00–2.75)	**0.005**
Yes	13	2.85 (2.65–2.85)	
Pathological stage (pTNM stage)			
Stage I + II	112	2.35 (1.95–2.75)	**0.021**
Stage III + IV	98	2.65 (2.10–2.80)	

*Indicated statistical significance (*P* < 0.05)

^a^Median of relative expression, with 25th–75th percentile in parenthesis

## Discussion

Microrchidia (MORC) is a highly conserved nuclear protein family. MORC2 as a member of MORC family, ubiquitously expressed in human cells and tissues, which has been found to be involved in several nuclear functions including repression gene transcription and regulation chromatin remodeling during the DNA-damage response [16]. In addition, MORC2 was shown to exert its cytosolic function in lipogenesis and adipogenic differentiation [19]. Our recent research found an association between MORC2 and gastric cancer [20, 21]. In this study, we demonstrate that MORC2 interacts with C/EBPα and promotes the sumoylation of C/EBPα and its subsequent degradation, which explores a different mechanism from previous studies to MORC2 function.

Previous studies have implied that muscle cell formation occurs at low C/EBPα levels while adipocyte-specific differentiation requires maximum C/EBPα expression [33]. The multipotent C2C12 mesenchymal progenitor cells can differentiate into adipocyte, muscle cells and osteoblasts. The decision of adipocyte or muscle cell differentiation is involved the expression level of C/EBPα [26, 27]. Here, we examined the role of MORC2 in cell differentiation using the C2C12 cells. Upon treatment with containing 2% horse serum DM, compared to differentiated control cells (LV-NC-C2C12), the ectopic expression of MORC2 in C2C12 cells impaired muscle cells differentiation, and indicated that dramatically down-regulated the expression level of C/EBPα. Interestingly, recent study indicated that the knockdown of MORC2 in 3T3-L1cells inhibited adipocyte differentiation upon stimulation with the adipocyte differentiation, and showed the decreased expression level of C/EBPα [19], suggesting that MORC2 up-regulate C/EBPα expression and promote adipogenic differentiation in 3T3-L1cells by promoting ACLY activity. These data indicate that, in different cell types and different stimulation, MORC2 may either down-regulate C/EBPα expression to impair muscle differentiation, or can up-regulate C/EBPα expression to promote adipocyte differentiation, suggesting that MORC2 may modulate the C/EBPα-mediated differentiation control switch to decide cell fate. The dual-regulated expression of C/EBPα by MORC2 enables precise insight into combinatorial effects of two key differentiation factors involved in differentiation control switch. Although, in our study, we used the mouse C2C12 cell line and cancer cell lines to study MORC2 involved in cell differentiation and proliferation, we can draw a conclusion that the over-expression of MORC2 can inhibit cell differentiation and promote cell proliferation.

Thus, based on our novel findings, we propose a model (Fig. 6h) for the role of MORC2 controlling C/EBPα expression via sumoylation to regulate the switch from a cell differentiation (Fig. 6h–a) to a cell proliferation (Fig. 6h–b) program and induce the tumorigenesis. In this study, we found that the overexpression of MORC2 down-regulated the C/EBPα expression via sumoylation modification to inhibit differentiation and resulted in gastric tumorigenesis. We can draw a conclusion that MORC2 inhibits C/EBPα-mediated signal pathway which involves in the switch from a cell differentiation to a cell proliferation program in cancer.

Recent studies show *morc2* mutations are absent in some diseases and cancer patients [28–32], implicating the emerging importance of MORC2 in human disease and cancers. Importantly, our results indicated that overexpression of MORC2 associated with poor differentiation status of gastric cancer, tumor size, depth of invasion, distant metastasis, pathological stage and 5-year survival rate in clinical gastric cancer, suggesting that MORC2 may be involved in progression and prognosis of gastric cancer. Meanwhile, the result also suggest that MORC2 may have a relation to metastasis in clinical gastric cancer which will be studied in our future. Therefore, evidence is mounting in implicating MORC2 as an oncogene. Further study of MORC2 may provide promising new therapeutic targets for gastric cancer.

## Methods

### Cell cultures, differentiation assays, and lentiviral productions

HEK-293T cells, gastric cancer SGC-7901 and BGC-823 cells and breast cancer MCF-7 cells, mouse C2C12 cells were maintained in Dulbecco’s modified Eagle’s medium (DMEM, GIBCO) supplemented with 10% FBS,100 U/ml penicillin and 100 μg/ml streptomycin at 37 °C in 5% CO_2_. C2C12 cells was induce differentiation from GM to DM for 7 days, the procedures were performed according to the previously described [22]. Recombinant MORC2-lentivirus including shRNA-MORC2, overexpression of LV-MORC2, LV- C/EBPα-WT, LV-C/EBPα-K161R, and LV-control vectors were purchased from Shanghai GeneChem Company. According to the manufacturer’s protocol, cells were infected with lentivirus in 12-well dishes in the presence of polybrene (4 g/mL). Selection with puromycin (2 g/mL) was started 48 h after lentiviral transduction to product stable expression MORC2. Infected cells were identified by Western blot.

### Plasmid construction, mutagenesis, and cell transfection

His-pcDNA3.1-C/EBPα and pGEX-2T-C/EBPα plasmids were generously provided by Drs. Friedman AD [33] and Dr. Smola-Hess S [34]. T7-SUMO1 expression plasmid was kindly gifted by Hu [35]. C/EBPa Double Nickase plasmid (h) was ordered from Santa Cruz company. The human full length and truncated versions of pcDNA-his-MORC2 were generated as described previously [17]. The Flag-MORC2 and GST-MORC2 vectors were acquired by KpnI and XhoI using the pcDNA3.1-MORC2 as template. The truncations of C/EBPα were cloned into the His-pcDNA3.1A vector. Mutation on K161R was generated using the Quickchange-XL Site-Directed Mutagenesis kit (Stratagene) from the His-C/EBPα plasmid of full length. The specific PCR primers are shown in Supplementary Table 1. Cells were transfected with siRNA and plasmid vectors using Lipofectamine 2000 (Invitrogen).

### Reverse transcription and quantitative real-time PCR

Total cellular RNA was extracted using TRIzol (Tiangen) according to the manufacturer’s protocol. One microgram of total RNA was reverse transcribed to cDNA in a total volume of 20 µl system using a RT reaction kit (Tiangen). Quantitative Real-time PCR was performed using a Mx 3000P real-time PCR system (Applied Biosystems) according to the manufacturer’s instruction and SYBR^®^ Premix Ex Taq (TaKaRa) as a DNA-specific fluorescent dye. PCR was carried out for 50 cycles of 95 °C for 10 s and 60 °C for 30 s. The specific PCR primers are shown in Supplementary Table 2. All the reactions were repeated at least three times. Gene expression levels were calculated relative to the housekeeping gene GAPDH by using Stratagene Mx 3000 P software.

### GST pull-down assay, Western blot, and immunoprecipitation assay

In vitro transcription and translation of His-MORC2 or Flag-C/EBPα proteins were performed by using the TNT-coupled transcription and translation system (Promega), as previously described [20]. The procedures of western blot and Immunoprecipitation assays were performed as previously described [17]. Primary antibody dilution and order company of His (1:2000, GenScript Corporation), c-myc and sumo1 (1:1000, Santa Cruz) and C/EBPα and Ubiquitin(1:1000, Cell Signaling Technology); Flag (1:2000, Shang Hai, Genomics); MORC2 (1:2000, Abcam);TFF1(1:1000, Protein tech Group).

### Confocal imaging

SGC-7901 cells co-transfected with His-C/EBPα together with Flag-vector or Flag-MORC2 grown on glass coverslips were fixed in methanol at room temperature for 15 min, and blocked with normal goat serum for 1 h. The cells were incubated with mouse anti-Flag-tagged antibody and rabbit anti-C/EBPα antibody for overnight at 4 °C, and washed three times in PBST (PBS with 1‰ TritonX-100).Then incubated with mouse anti-goat Alexa-594 and rabbit anti-goat Alexa-488 secondary antibodies (1:100, Molecular Probes) for 1 h at room temperature, washed three times in PBST. The DNA dye DAPI was used to co-stain the DNA (blue). Confocal scanning analysis was performed by using a Leika laser confocal scanning microscope in accordance with established methods, utilizing sequential laser excitation to minimize the possibility of fluorescent emission bleed-through.

### Cell cycle analysis, growth curve, and colony formation assay

Stable overexpressed cell lines were seeded in 60-mm plates to perform the flow cytometry and cell counts assays as described in our previous paper [17, 20]. For colony formation assay, 500 cells were plated in six-well plates to assess the proliferation potential of cells and incubated at 37 °C in a 5% CO_2_ incubator. After 2 weeks, the number of colonies was counted. Data represent the mean ± SD from three independent experiments performed in triplicate wells

### Mouse models

The Ethics Committee of China Medical University approved the study protocol for the use of experimental animals. Female Balb/c nude mice (18–22 g) were purchased from Beijing Vital River Laboratory Animal Technology Company (Beijing, China). To assess role of MORC2 in breast tumor progression, each mouse was injected subcutaneously with 2 × 10^6^ cells in 0.2 ml PBS into right scapular region. Four groups (5 each) of mice were injected MCF-7 cells with lentivirus-mediated stable expression of MORC2 and control vector including shRNA-MORC2, respectively. Tumor size was measured every 2 days with calipers. The tumor volume was calculated based on the formula (*L* × *W*^2^)/2, where *L* is length and *W* is width. Mice were sacrificed after 6 weeks, and the xenograft volume was monitored by weight.

### Tumor tissue samples and immunohistochemistry

Samples of human gastric cancer tissues, paired-adjacent non-tumor gastric tissues and paraffin-embedded gastric tumor tissues were obtained from the First Hospital of China Medical University, as previously described [20]. Fresh samples were rapidly frozen in liquid nitrogen immediately after resection and stored at –80 ℃. All samples were obtained with patients’ informed consent and were histologically confirmed by staining with hematoxylin-eosin. The histological grade of cancers was assessed according to criteria set by the World Health Organization. All research involving human participants have been approved by the First Hospital of China Medical University ethics committees. The Immunohistochemistry procedures and intensity of images were analyzed as previously described [20, 21].

### Statistical analysis

Quantitative data are presented as mean values ± SEM (standard error of the mean) from≥3 independent repetitions. Statistical comparisons between groups were carried out with the use of 2-tailed Student *t* test. A *p*-value of *p* < 0.05 was considered as statistically significant.

## Supplementary information

MORC2 regulated C/EBPα expression and affected its stability

Expression of MORC2 in C2C12 cells was confirmed

The effect of MORC2 on tumorigenesis with MCF-7 cells

Description of Supplementary Figure

Primers used for construction of the full length and truncation of C/EBPα with Flag-tag and GST-tag

Primer Sequences were used for quantitative Real-Time PCR analysis
