# Supplementary figures and images for: MORC2 regulates C/EBPα-mediated cell differentiation via sumoylation
Source: Cell Death Differ. 2019 Jan 15;26(10):1905–17. doi: 10.1038/s41418-018-0259-4 (PMC6748086; doi:10.1038/s41418-018-0259-4)

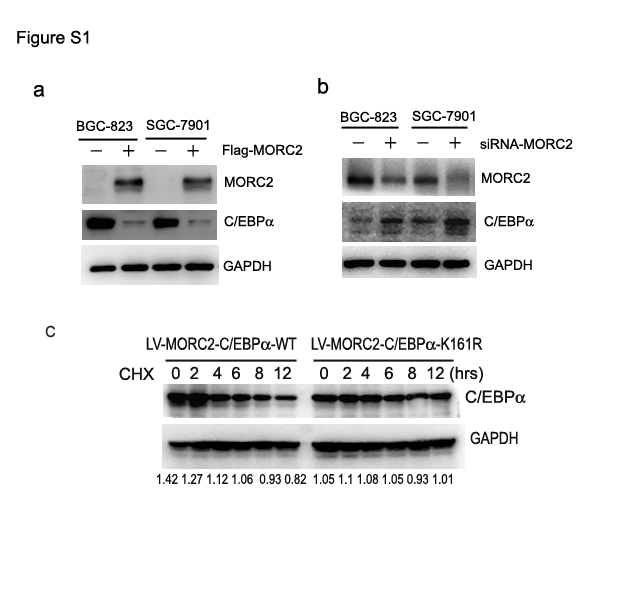

Supplement: Supplementary file 1 — MORC2 regulated C/EBPα expression and affected its stability [file 41418_2018_259_MOESM1_ESM.tif]

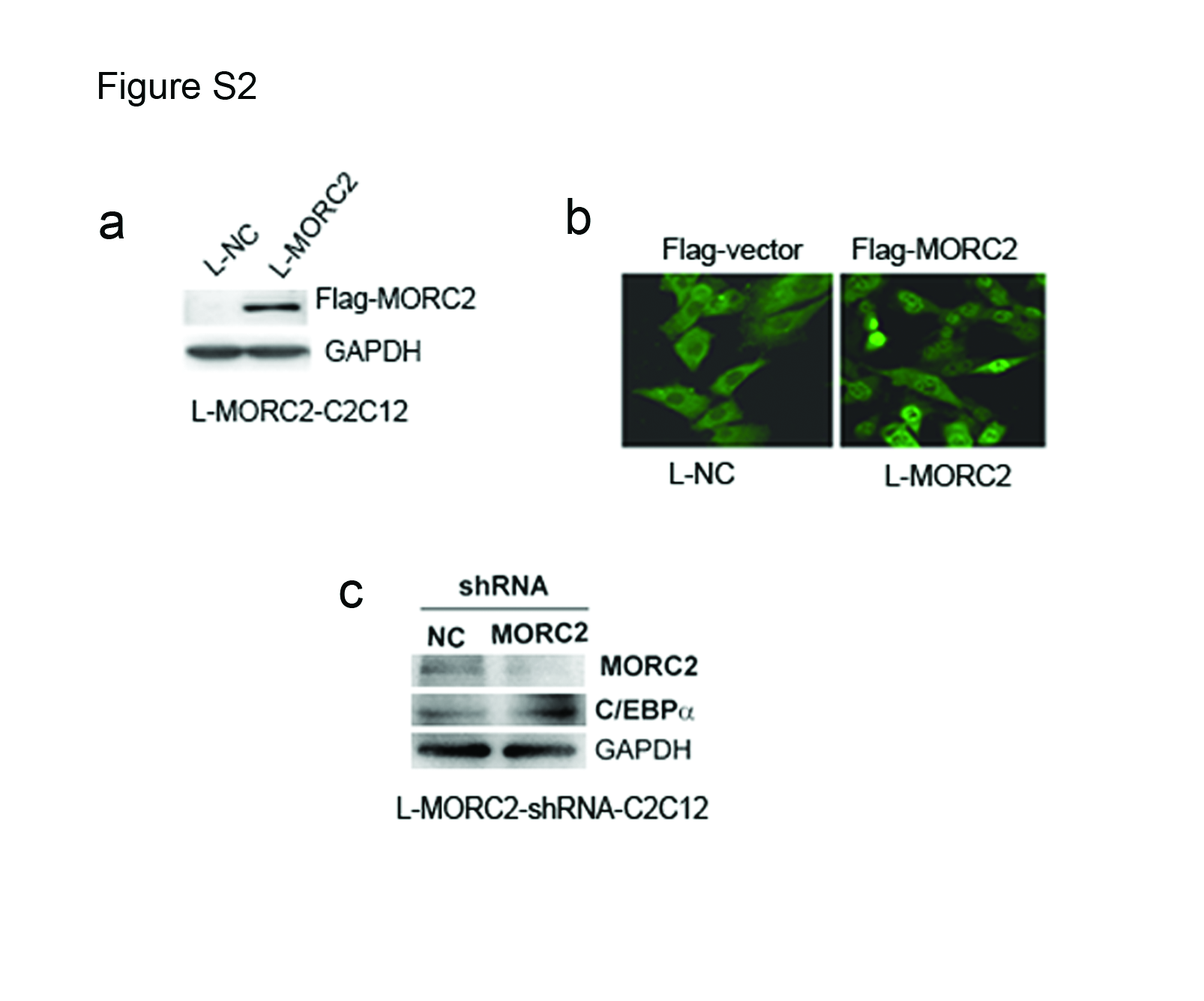

Supplement: Supplementary file 2 — Expression of MORC2 in C2C12 cells was confirmed [file 41418_2018_259_MOESM2_ESM.tif]

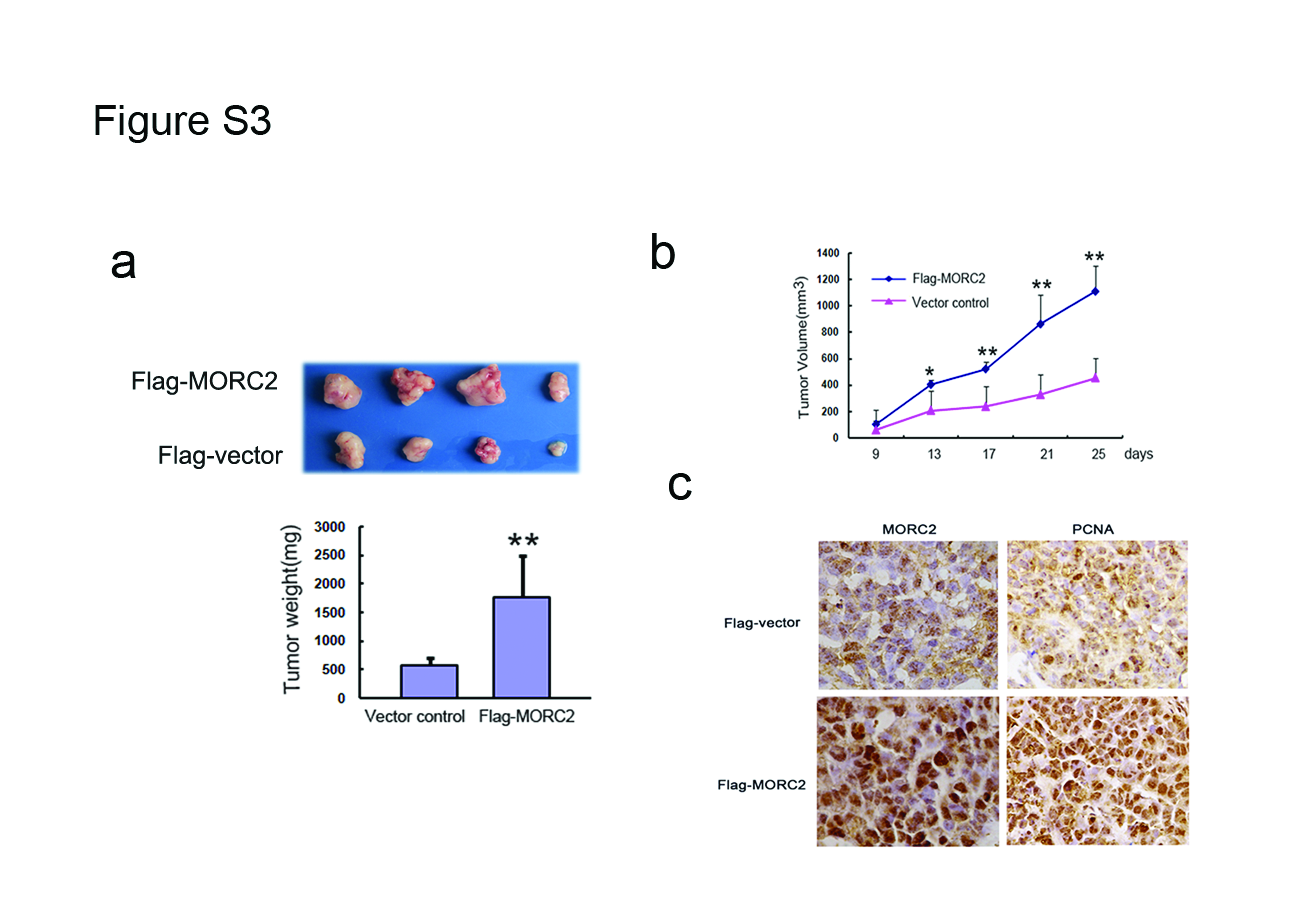

Supplement: Supplementary file 3 — The effect of MORC2 on tumorigenesis with MCF-7 cells [file 41418_2018_259_MOESM3_ESM.tif]
