# Supplementary material for: MORC2 regulates C/EBPα-mediated cell differentiation via sumoylation
Source: Cell Death Differ. 2019 Jan 15;26(10):1905–17. doi: 10.1038/s41418-018-0259-4 (PMC6748086; doi:10.1038/s41418-018-0259-4)
Supplement: Supplementary file 4 — Description of Supplementary Figure [file 41418_2018_259_MOESM4_ESM.docx]

**Description of Supplementary Figure1**

1. Western blot results shown that overexpression of MORC2 down-regulated C/EBPα expression in BGC-823 cells and SGC-7901 cells.
2. Western blot results shown that Knock down MORC2 up-regulated C/EBPα expression in SGC-7901 cells and BGC-823 cells.
3. The protein levels of C/EBPα-K161R was more stability than C/EBPα-WT with increasing CHX treatment time.

**Description of Supplementary Figure2**

(a) The expression level of ectopic MORC2 was determined by Western blot. C2C12 cells were infected with lentiviral-Flag-MORC2 for 72h. Lentiviral-Flag-vector was used as a negative control. The cells were screened with puromycin (1μg/ml). Total lysate from C2C12 cells undergoing differentiation was prepared on the days indicated and analyzed by immunoblotting with anti-Flag and anti-GAPDH antibodies.

(b) The infection efficiency of C2C12 cells was determined by confocal microscopy with anti-Flag antibody. Alexa Fluor 488(green) was used to detect anti-Flag antibody.

(c) Knockdown of MORC2 up-regulates C/EBPα expression in C2C12 cells by western blot. C2C12 cells were infected with either Lentivirus MORC2-shRNA vector (MORC2-shRNA) or Lentivirus NC-shRNA control vector (NC-shRNA) and transferred the cells to the differentiation medium. The ability of MORC2-shRNA to knockdown the expression of MORC2, and expression of C/EBPα in C2C12 cells was confirmed by western blot.

**Description of Supplementary Figure3**

(a) MCF-7 cells (without endogenous C/EBPα expression) stably expressing Flag-vector or Flag-MORC2 were injected into nude mice (n=6 per group). The inoculated mice was terminated in 3 weeks. Each tumor lump was removed from the body. Photographs of tumor weight was quantiﬁed.

(b)Tumor volumes were measured and data are mean±SEM.

(c) Immunohistochemistry staining of anti-MORC2 in nude mice tumor tissues sections, Original magniﬁcation, ×400. Degree of intratumoral proliferation was determined by PCNA staining.
