# Supplementary material for: MORC2 regulates C/EBPα-mediated cell differentiation via sumoylation
Source: Cell Death Differ. 2019 Jan 15;26(10):1905–17. doi: 10.1038/s41418-018-0259-4 (PMC6748086; doi:10.1038/s41418-018-0259-4)
Supplement: Supplementary file 5 — Primers used for construction of the full length and truncation of C/EBPα with Flag-tag and GST-tag [file 41418_2018_259_MOESM5_ESM.doc]

**Supplementary Table 1**

Primers used for construction of the full length and truncation of C/EBPα with Flag-tag and GST-tag

| C/EBPα | primers |
| --- | --- |
| Full length | F:CTAAAGCTTACCGGTCCGGAATTCATGGAGTC |
|  | R: CTTGGATCCTCACGCGCAGTTGCCCAT |
| C1(1-221) | F: TACCGGTCCGGAATTCAT GG AGTC |
|  | R: CTACTCGAGCTGCAGGTGCATGGTGGTC |
| C2(103-359) | F: CGGAATTCGGCGGCGACTTTGACTAC |
|  | R: CTACTCGAGCCTCG ACTCACGCGCAGTTG |
| C3(1-108) | F: TACC GGTCCGGAATTCATGGAGTC |
|  | R: CTACTCGAGGGTAGTCA AAGTCGCC |
| C4(103-212) | F: CGGAATTC GGCGGCGACTTTGACTAC |
|  | R: CATCTCGAG TGCGCGATCTGGAAC |
| C5 (218-359) | F: CGGAATTC ATGCACCTGCAGCCCGGTCAC |
|  | R: CTACTCGAG CCTCGACTCACGCGCAGTTG |
| C/EBPα-K161R | F**:** 5’-CCGCTGGTGATCAGGCAGGAGCCCGC-3’ |
|  | R: 5’-GGGGCTCCTGCCTGATCACCAGCGGG-3’ |
