# Supplementary material for: MORC2 regulates C/EBPα-mediated cell differentiation via sumoylation
Source: Cell Death Differ. 2019 Jan 15;26(10):1905–17. doi: 10.1038/s41418-018-0259-4 (PMC6748086; doi:10.1038/s41418-018-0259-4)
Supplement: Supplementary file 6 — Primer Sequences were used for quantitative Real-Time PCR analysis [file 41418_2018_259_MOESM6_ESM.doc]

**Supplementary Table 2**

Primer Sequences were used for quantitative Real-Time PCR analysis

| genes | Primer sequences |
| --- | --- |
| c-myc | F: 5’-GCCTCAGAGTGCATCGAC-3’ |
|  | R: 5’-TCCACAGAAACAACATCG-3’ |
| TFF1 | F: 5’-TTGTGGTTTTCCTGGTGTCA-3’ |
|  | R: 5’-CCGAGCTCTGGGACTAATCA-3’ |
| MyoD | F: 5’-GCCTGAGCAAAGTGAATGAG-3’ |
|  | R: 5’-CTTCGATGTAGCGGATGG-3’ |
| Myogenin | F: 5’-AGCCACACTGAGGGAGAAG-3’ |
|  | R: 5’-GTTGAGGGAGCTGAGCAAG-3’ |
| MHC | F: 5’-TGAACTGGAGGGTGAGGTAG-3’ |
|  | R: 5’-TTCGGTCTTCTTCTGTCTGG-3’ |
| MORC2 | F: 5’-GCAAGCGGGGCAGATTT-3’ |
|  | R: 5’-CTTGGTGTCGTGTCTGTGGG-3’ |
| GAPDH | F**:** 5’- GAAGGTCGGAGTCAACGGAT-3’ |
|  | R: 5’- CTGGAAGATGGTGATGGGATT-3’ |

NOTE. Glyceraldehyde-3-phosphate dehydrogenase (GAPDH) was amplified as an internal control.
